# Anti-Inflammatory and Antinociceptive Activities of Anthraquinone-2-Carboxylic Acid

**DOI:** 10.1155/2016/1903849

**Published:** 2016-01-03

**Authors:** Jae Gwang Park, Seung Cheol Kim, Yun Hwan Kim, Woo Seok Yang, Yong Kim, Sungyoul Hong, Kyung-Hee Kim, Byong Chul Yoo, Shi Hyung Kim, Jong-Hoon Kim, Jae Youl Cho

**Affiliations:** ^1^Department of Genetic Engineering, Sungkyunkwan University, Suwon 440-746, Republic of Korea; ^2^Division of Gynecologic Oncology, Department of Obstetrics and Gynecology, Ewha Womans University Mokdong Hospital College of Medicine, Ewha Womans University, Seoul 158-710, Republic of Korea; ^3^Colorectal Cancer Branch, Research Institute, National Cancer Center, Goyang 410-769, Republic of Korea; ^4^Department of Veterinary Physiology, College of Veterinary Medicine, Biosafety Research Institute, Chonbuk National University, Iksan 54596, Republic of Korea

## Abstract

Anthraquinone compounds are one of the abundant polyphenols found in fruits, vegetables, and herbs. However, the* in vivo* anti-inflammatory activity and molecular mechanisms of anthraquinones have not been fully elucidated. We investigated the activity of anthraquinones using acute inflammatory and nociceptive experimental conditions. Anthraquinone-2-carboxylic acid (9,10-dihydro-9,10-dioxo-2-anthracenecarboxylic acid, AQCA), one of the major anthraquinones identified from Brazilian taheebo, ameliorated various inflammatory and algesic symptoms in EtOH/HCl- and acetylsalicylic acid- (ASA-) induced gastritis, arachidonic acid-induced edema, and acetic acid-induced abdominal writhing without displaying toxic profiles in body and organ weight, gastric irritation, or serum parameters. In addition, AQCA suppressed the expression of inflammatory genes such as cyclooxygenase- (COX-) 2 in stomach tissues and lipopolysaccharide- (LPS-) treated RAW264.7 cells. According to reporter gene assay and immunoblotting analyses, AQCA inhibited activation of the nuclear factor- (NF-) *κ*B and activator protein- (AP-) 1 pathways by suppression of upstream signaling involving interleukin-1 receptor-associated kinase 4 (IRAK1), p38, Src, and spleen tyrosine kinase (Syk). Our data strongly suggest that anthraquinones such as AQCA act as potent anti-inflammatory and antinociceptive components* in vivo*, thus contributing to the immune regulatory role of fruits and herbs.

## 1. Introduction

It is well known that chronic inflammation causes serious diseases such as cancer and diabetes, thereby affecting the death rate of humans [1]. Molecular and cellular factors involved in these pathophysiological phenomena include the generation of free radicals, inflammatory gene expression, and recruitment of inflammatory cells [2, 3]. Because of serious concerns over the role of inflammation in human health and longevity, in 2004, the magazine Time stressed chronic inflammation as a secret killer. As a consequence, the prevention of chronic inflammation has become an important issue. One well-known approach to decreasing unexpected and long-lasting inflammation includes ingestion of antioxidants to protect against oxidative stress [4]. In addition, to suppress the body's inflammatory responses, special remedies with anti-inflammatory activity are also becoming available. For these purposes, people are recommended to consume numerous functional foods and nutraceuticals such as taheebo, ginseng, mushrooms, and cordyceps, as well as fresh vegetables and fruits [5–7]. These are all great sources of valuable components with antioxidative, antiasthmatic, and anti-inflammatory properties such as flavonoids, saponins, alkaloids, terpenoids, and anthraquinones [8–11]. Indeed, in many different experiments with animal models, these foods have been demonstrated to display numerous beneficial pharmacological activities.

**Figure 1:**
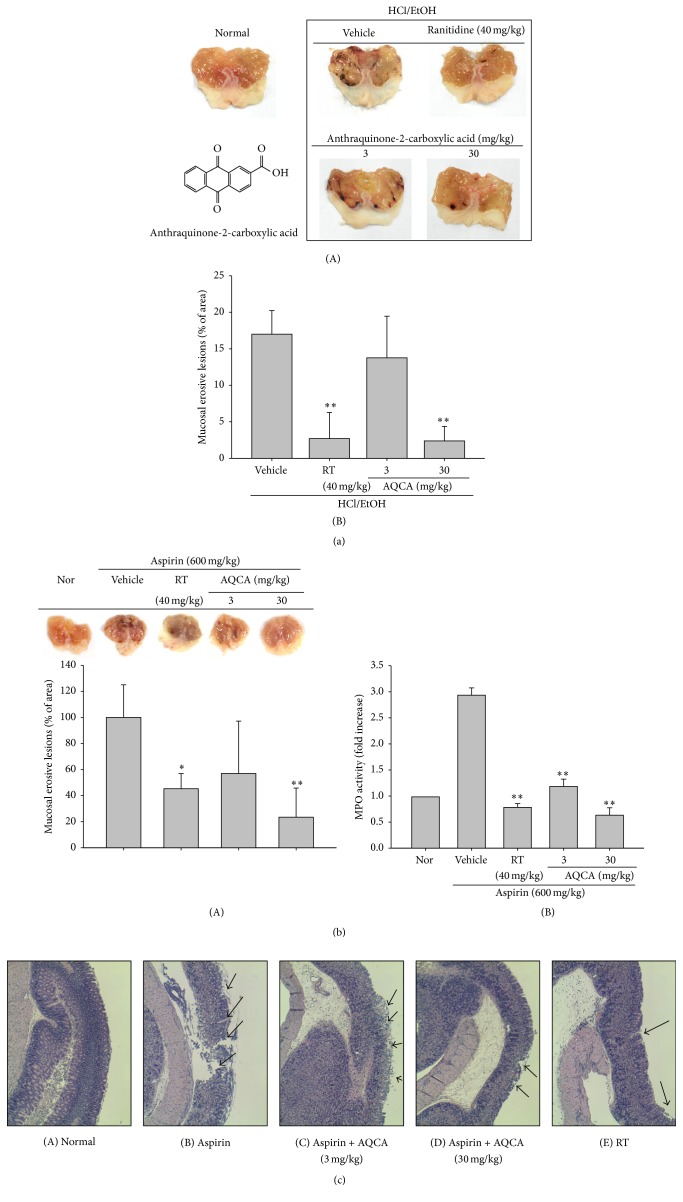
Effect of AQCA on* in vivo* inflammatory symptoms. ((a)(A) and (b)(A)) Mice were treated with orally administered AQCA (0, 3, and 30 mg/kg) or ranitidine (40 mg/kg) for 3 days prior to oral administration of EtOH/HCl or aspirin (600 mg/kg). After 24 h, gastric lesions in the stomach were measured using a pixel counter ((a)(B) and (b)(B)), and photographs of these lesions were taken using a camera ((a)(A) and (b)(A)). ((b)(B)) MPO activity, indicative of neutrophil infiltration in the stomach, was determined in total lysates of stomach from aspirin-treated mice administered with AQCA. (c) Histological examination of aspirin-treated stomach was performed by H&E staining after parafilm-fixed section. ^*∗*^ *P* < 0.05 and ^*∗∗*^ *P* < 0.01 compared with the control group.

Among the valuable compounds present in food, anthraquinones have been the focus of our research on understanding the anti-inflammatory role in the human body and their inhibitory mechanism. Unlike other active ingredients such as flavonoids, the action of these compounds has not been fully elucidated, even though these they are prominently found in nature. Several reports have claimed that some anthraquinones, such as anthrasesamones (A, B, C, and D), catenarin, emodin, rhein, physcion, and chrysophanol, display anti-inflammatory activities by suppressing nuclear factor- (NF-) *κ*B and activator protein- (AP-) 1 activation pathways in various cell systems such as macrophages, hepatic stellate cells, fibroblasts, and glioma cells [12–15]. Although numerous studies have reported their anti-inflammatory activities* in vitro*, anthraquinones function in* in vivo* inflammatory models has not been fully examined. Therefore, in the present study, we aimed to investigate the* in vivo* efficacy of anthraquinone-2-carboxylic acid (AQCA, Figure 1(a)), a representative anthraquinone-type compound, derived from taheebo. Using inflammatory and algesic animal models mimicking the symptoms of human gastritis, edema, and abdominal pain, we explored the therapeutic potential of orally administered AQCA and its anti-inflammatory mechanism.

## 2. Materials and Methods

### 2.1. Materials

Anthraquinone-2-carboxylic acid (AQCA, Figure 1(a)), indomethacin (Indo), ranitidine (RT), arachidonic acid, sodium carboxyl methylcellulose (Na CMC), phorbol-12-myristate (PMA), acetylsalicylic acid (ASA), and lipopolysaccharide (LPS,* E. coli* 0111:B4) were purchased from Sigma Chemical Co. (St. Louis, MO, USA). SB203580 (SB), PP2, and piceatannol (Picea) were obtained from Calbiochem (La Jolla, CA, USA). All other chemicals used in this study were of analytical grade from Sigma Chemical Co. Phosphospecific or total antibodies that were raised against Src (Cat. numbers 2101 and 2102), spleen tyrosine kinase (Syk) (Cat. numbers 2711 and 2712), p38 (Cat. numbers 4631 and 9212), c-Jun N-terminal kinase (JNK) (Cat. numbers 9251 and 9252), cyclooxygenase- (COX-) 2 (Cat. number 4842), interleukin-1 receptor-associated kinase 4 (IRAK4) (Cat. number 4363), and *β*-actin (Cat. number 4967) were obtained from Cell Signaling (Beverly, MA, USA). RAW264.7 and HEK293 cells were purchased from American Type Culture Collection (Manassas, VA, USA). Myeloperoxidase (MPO) activity colorimetric assay kit was obtained from Biovision (Milpitas, CA, USA). Luciferase constructs that contained the binding promoters for NF-*κ*B and AP-1 were gifts from Professor Hae Young Chung (Pusan National University, Pusan, Korea) and Addgene (Cambridge, MA, USA).

### 2.2. Mice

Male ICR mice, 6 weeks old, and Balb/c mice, 7 weeks old, were purchased from DAEHAN BIOLINK (Chungbuk, Korea) and were housed in groups of 6–8 mice under a 12 h light/dark cycle (lights on at 6 a.m.). Water and pellet diets (Samyang, Daejeon, Korea) were supplied* ad libitum*. Animals were cared for in accordance with the guidelines issued by the National Institute of Health for the Care and Use of Laboratory Animals (NIH Publication 80-23, revised in 1996). Studies were performed in accordance with guidelines established by the Institutional Animal Care and Use Committee at Sungkyunkwan University (Suwon, Korea; approval ID: SKKUBBI 13-6-6).

### 2.3. Cell Culture

RAW264.7 cells, a murine macrophage cell line, peritoneal macrophages, and human embryonic kidney 293 (HEK293) cells were maintained in RPMI1640 media supplemented with 100 U/mL penicillin, 100 *μ*g/mL streptomycin, and 10% FBS. Cells were grown at 37°C and 5% CO_2_ in humidified air.

### 2.4. Drug Preparation

The stock solutions of AQCA used in the* in vivo* experiments were prepared using 0.5% Na carboxymethylcellulose (CMC). Mice administered with or without inflammation-inducing agents (control or normal groups) were orally treated with 0.5% Na CMC alone by gavage. For* in vitro* study, this compound was dissolved with 100% dimethyl sulfoxide (DMSO).

### 2.5. EtOH/HCl- and Aspirin-Induced Gastritis

Gastric lesions were induced with EtOH/HCl and ASA (600 mg/kg), according to a published method [16]. Fasted ICR mice (*n* = 7) were orally treated with AQCA (3 and 30 mg/kg) or ranitidine (40 mg/kg) three times every 8 h in a day. Thirty minutes after the last administration, 400 *μ*L of 60% ethanol in 150 mM HCl was injected orally. Each animal was anesthetized with an overdose of urethane 1 h after the administration of necrotizing agents. The stomachs were then excised and gently rinsed under running tap water. The stomachs were opened along the greater curvature and spread out on a board, and the area (mm^2^) of mucosal erosive lesions was measured using a pixel-counter. The establishment of aspirin-induced gastric ulcers followed a previously published method [17]. Fasted ICR mice (*n* = 5) were treated with either AQCA (3 and 30 mg/kg) intragastrically. After 30 minutes, mice were given orally administered aspirin (600 mg/kg) and were left attended for 3 hours before sacrifice. Each animal was anesthetized with an overdose of urethane 3 h after the administration of necrotizing agents. The stomachs were then excised and gently rinsed under running tap water.

### 2.6. Histological Analysis of Aspirin-Treated Stomach

Tissue samples taken from the stomachs of the mice at 8 h after challenge with aspirin (600 mg/kg) were fixed with 10% formalin in PBS and then embedded in paraffin. Approximately 4 *μ*m thin tissue sections were stained with haematoxylin and eosin for histopathological examination as reported previously [18].

### 2.7. MPO Assay

MPO activity was measured using an MPO activity colorimetric assay kit. Stomach and pancreas were homogenized in 4 volumes of PBS containing 0.1% NP40. After lysis, samples were centrifuge at 13000 ×g for 10 min to remove insoluble material. Soluble materials were diluted in MPO assay buffer. MPO substrate was added, samples were incubated at 25°C for 60 min, and then stop mix was added. Incubation was continued for an additional 10 min to stop the reaction. TNB (trinitrobenzene sulfonic acid) reagent containing the DTNB [5,5′-dithiobis-(2-nitrobenzoic acid)] probe and TCEP [Tris(2-carboxyethyl)phosphine hydrochloride] was added. The presence of MPO was measured at 412 nm. MPO activity was expressed as U/g tissue. One unit of MPO activity was defined as the amount of MPO which generates sufficient taurine chloramines to consume 1 *μ*M TNB/min at 25°C.

### 2.8. Arachidonic Acid-Induced Mouse Algesic Model

To test whether AQCA is able to ameliorate algesic property induced by arachidonic acid, as reported previously [19, 20], ICR mice (*n* = 7) were orally pretreated with AQCA (3 and 30 mg/kg) or indomethacin (5 mg/kg) for 7 days. After the final treatment, arachidonic acid (2% (w/v)) was applied to the ear of the mouse (25 *μ*L/ear) as described previously [21]. The thickness of the oedema was measured with a constant-pressure thickness gauge 1 h after treatment with arachidonic acid. To evaluate hypoalgesic efficacy, the inhibitory effect of AQCA on ear oedema was calculated as follows:

(1) %  of  controloedema  inducer  alone=DET−NETCET−NET×100,

where D_ET_ is ear thickness of drug-treated group, N_ET_ is ear thickness of normal untreated group, and C_ET_ is ear thickness of control group.

### 2.9. Acetic Acid-Induced Abdominal Writhing

ICR mice (*n* = 7/group) were orally treated with AQCA (0, 3, 30, and 60 mg/kg) or indomethacin (10 mg/kg). Following the 60 min treatment period, mice were intraperitoneally injected (i.p.) with acetic acid (5%), and 5 min later, the number of abdominal writhes was recorded for a period of 20 min.

### 2.10. Acute Toxicity Test and Measurement of Serum Parameters

Three mice were orally administered with AQCA (1 g/kg) or aspirin (300 mg/kg) for 7 days. Then, mortality and the change of body weights were monitored daily until finished. All animals from each group were then sacrificed and the weight of key organs and gastric ulcer formation were examined. Serum samples were obtained by the centrifugation of blood at 4,000 rpm for 15 min at 4°C and were then divided into Eppendorf tubes. Isolated sera were stored at −20°C until they were used for the analyses. The levels of serum alanine aminotransferase (ALT), aspartate aminotransferase (AST), and cholesterol were measured with a Roche Modular spectrophotometric autoanalyzer.

### 2.11. mRNA Analysis by Semiquantitative or Quantitative Reverse Transcriptase-Polymerase Chain Reaction

To determine inflammatory gene expression levels, total RNA was isolated from stomach tissues or LPS-treated RAW264.7 cells in the presence or absence of AQCA or ranitidine with TRIzol Reagent (Gibco), according to the manufacturer's instructions. The RNA was stored at −70°C until use. mRNA quantification was performed by real-time RT-PCR with the SYBR Premix Ex Taq (Takara Bio Inc., Shiga, Japan), according to the manufacturer's instructions, and a real-time thermal cycler (Bio-Rad, Hercules, CA, USA) as reported previously [22]. Semiquantitative RT-PCR reactions were conducted as previously reported with minor modifications. The results were expressed as the ratio of the optimal density relative to GAPDH. The primers were obtained from Bioneer (Daejeon, Korea) and are described as in Table 1.

### 2.12. Preparation of Tissue/Cell Lysates and Immunoblotting

Stomach tissues or RAW264.7 cells were washed 3 times in cold PBS containing 1 mM sodium orthovanadate and then lysed in lysis buffer (20 mM Tris-HCl, pH 7.4, 2 mM EDTA, 2 mM ethyleneglycotetraacetic acid, 50 mM *β*-glycerophosphate, 1 mM sodium orthovanadate, 1 mM dithiothreitol, 1% Triton X-100, 10% glycerol, 10 *μ*g/mL aprotinin, 10 *μ*g/mL pepstatin, 1 mM benzimide, and 2 mM PMSF) for 30 min, with rotation, at 4°C. The tissue lysates were clarified by centrifugation at 16,000 ×g for 10 min at 4°C and stored at −20°C until needed.

Tissue or cell lysates were then analyzed using immunoblotting. Proteins (25 *μ*g/lane) were separated on 10% SDS-polyacrylamide gels and transferred by electroblotting to a polyvinylidene difluoride (PVDF) membrane. Membranes were blocked for 60 min at room temperature in Tris-buffered saline containing 3% FBS, 20 mM NaF, 2 mM EDTA, and 0.2% Tween 20. The membranes were incubated for 60 min with specific primary antibodies at 4°C, washed 3 times with the same buffer, and incubated for an additional 60 min with HRP-conjugated secondary antibodies. The total and phosphorylated levels of COX-2, Src, Syk, p38, IRAK4, and *β*-actin were visualized using an ECL system (Amersham, Little Chalfont, Buckinghamshire, UK), as reported previously [23].

### 2.13. Luciferase Reporter Gene Activity Assay

HEK293 cells (1 × 10^6^ cells/mL) were transfected with 1 *μ*g of plasmids containing NF-*κ*B-Luc or AP-1-Luc as well as *β*-galactosidase using the polyethylenimine (PEI) method in a 12-well plate according to a previous report [24]. Briefly, the transfected cells treated with AQCA in the presence or absence of PMA (20 ng/mL) were lysed in the culture dishes with reporter lysis buffer. Lysates were centrifuged at maximum speed for 10 min in an Eppendorf microcentrifuge. Ten *μ*L of the supernatant fraction was incubated with 50 *μ*L of luciferase substrate, and the relative luciferase activity was determined with a Luminoskan Ascent (Thermo Labsystems Oy, Helsinki, Finland). Luciferase activity was normalized to *β*-galactosidase activity.

### 2.14. Statistical Analyses

All data presented in this paper are expressed as the means ± SD of experiments. For statistical comparisons, the results were analyzed using either ANOVA/Scheffe's* post hoc* test or the Kruskal-Wallis/Mann-Whitney test. A *P* value < 0.05 was considered to be a statistically significant difference. All of the statistical tests were carried out using the computer program SPSS (SPSS Inc., Chicago, IL, USA). Similar experimental data were also obtained using an additional independent set of* in vivo* experiments which was conducted using the same numbers of mice.

## 3. Results and Discussion

**Figure 2:**
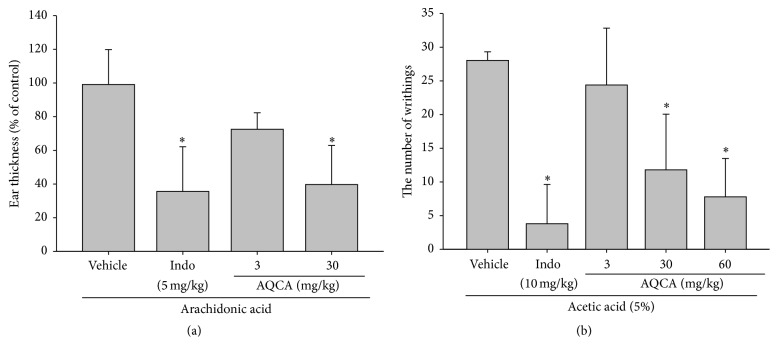
Effect of AQCA on* in vivo* nociceptive symptoms. (a) Effect of AQCA on arachidonic acid-induced ear edema was evaluated in ICR mice that were treated orally with AQCA (0, 3, and 30 mg/kg) or indomethacin (5 mg/kg) for 7 days and with topical application of 2% arachidonic acid solution (25 *μ*L/ear) to the left ear. The thickness of the edema was measured with a constant-pressure thickness gauge 1 h after treatment with arachidonic acid. The ear thickness of mice treated with the inducer alone was considered to be 100%. (b) Effect of AQCA on acetic acid-induced writhing was evaluated in ICR mice that were treated orally with AQCA (0, 3, 30, and 60 mg/kg) or indomethacin (10 mg/kg) for 3 days and intraperitoneally injected with 5% arachidonic acid solution. The number of writhings was recorded over 20 min and expressed as a percentage relative to the number of writhings in vehicle controls. ^*∗*^ *P* < 0.05 compared with the control group.

**Figure 3:**
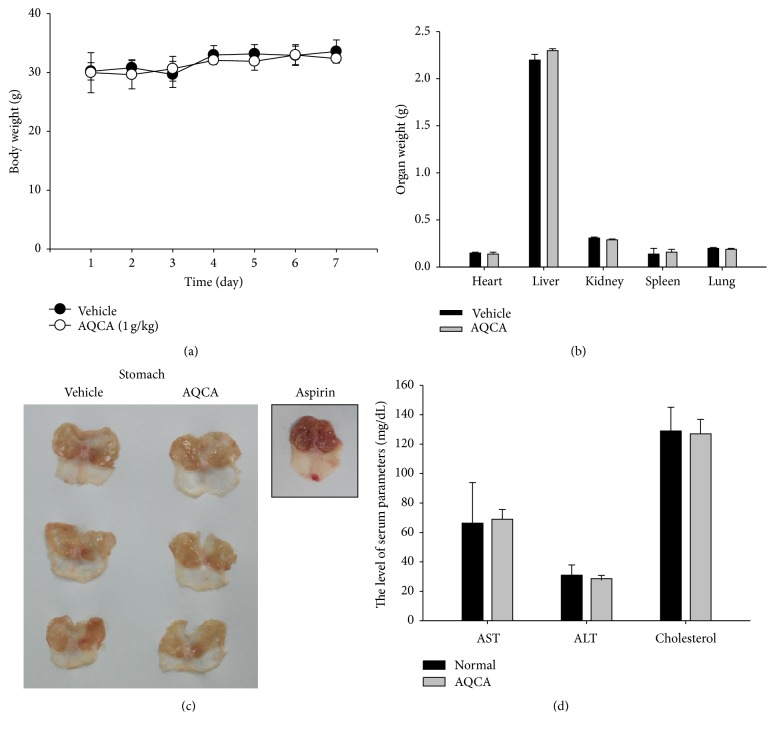
Acute toxicity of AQCA in mice. (a and b) Changes in total body weight or organ weight were evaluated following oral administration of AQCA for 7 days. (c) Formation of stomach lesions following oral administration of AQCA (1 g/kg) or aspirin (300 mg/kg) for 7 days. (d) Acute toxicity of oral administration of AQCA for 7 days was evaluated by measuring serum parameters (AST, ALT, and cholesterol). Data represent the mean ± SD of an experiment performed with three mice.

**Figure 4:**
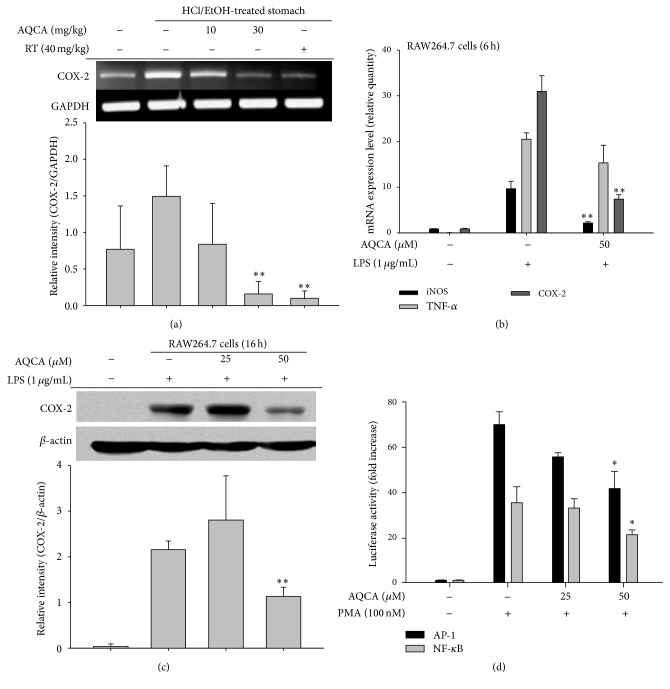
Effect of AQCA on the transcriptional activation of inflammatory genes. (a and b) mRNA levels of inflammatory genes (iNOS, TNF-*α*, and COX-2) in mice treated with HCl/EtOH or LPS-treated RAW264.7 cells were determined by semiquantitative (a) and real-time RT-PCR (b). (c) Total lysates were prepared from LPS-treated RAW264.7 cells treated with AQCA (25 and 50 *μ*M). The total forms of COX-2 and *β*-actin were analyzed by immunoblotting analysis. (d) Promoter binding activities of NF-*κ*B and AP-1 in the presence or absence of PMA were determined by reporter gene (luciferase) assay. Relative intensity (a and c) was calculated using level of GAPDH or *β*-actin and the DNR Bio-Imaging System (a and c bottom panels). ^*∗*^ *P* < 0.05 and ^*∗∗*^ *P* < 0.01 compared with the control group.

**Figure 5:**
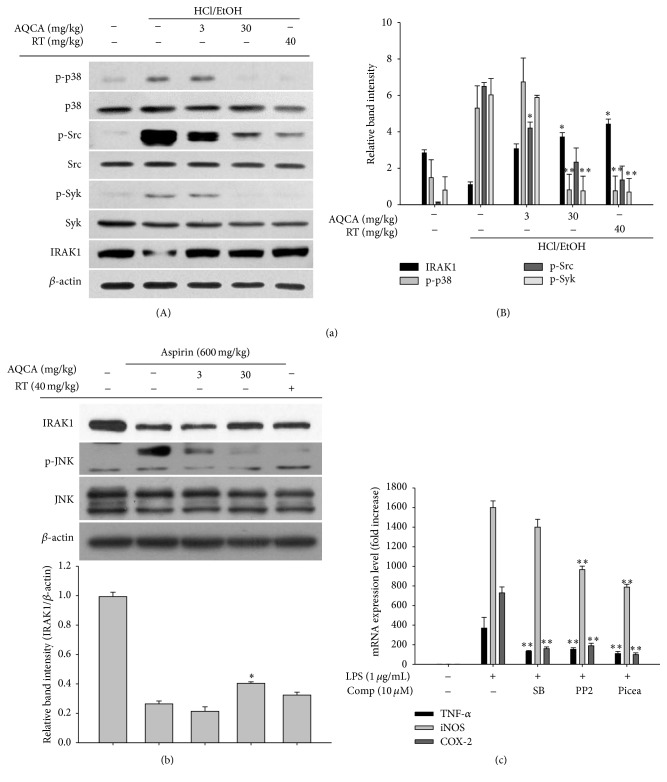
Effect of AQCA on the activation of signaling enzymes. (a and b) Total lysates were prepared from stomach tissues of mice treated with HCl/EtOH or aspirin that had been orally treated with AQCA (0, 3, and 30 mg/kg) or RT (ranitidine, 40 mg/kg). The total and phosphoforms of p38, JNK, Src, Syk, and IRAK1 were analyzed by immunoblotting analysis. (c) mRNA levels of inflammatory genes (iNOS, TNF-*α*, and COX-2) in LPS-treated RAW264.7 cells pretreated with SB203580 (SB), PP2, or piceatannol (Picea) were determined by real-time RT-PCR. Relative intensity was calculated using *β*-actin level and the DNR Bio-Imaging System ((a)(B) and (b) bottom panels). ^*∗*^ *P* < 0.05 and ^*∗∗*^ *P* < 0.01 compared with the control group.

Under our established conditions, we could observe a remarkable increase in gastric lesions and inflammatory cell (neutrophil) infiltration in HCl/EtOH- and aspirin-induced gastritis (Figures 1(a) and 1(b)). In addition, acute-phase nociceptive symptoms were clearly established in the arachidonic acid-induced ear edema (Figure 2(a)) and acetic acid-induced abdominal pain models (Figure 2(b)). To evaluate whether the curative activity of AQCA is independent of its toxicological profiles, toxicological parameters of body and organ weights, stomach irritation, and liver toxicity markers were measured (Figure 3). Furthermore, molecular analysis of inflammatory tissues revealed that COX-2 expression was markedly upregulated at the transcription and translational levels (Figures 4(a) and 4(c)) and that upstream signaling events for COX-2 expression, including the phosphorylation of p38, Src, JNK, and Syk and degradation of IRAK4, were also triggered by the treatment of HCl/EtOH or aspirin (Figures 5(a) and 5(b)).

As expected, orally administered AQCA (30 mg/kg) significantly ameliorated inflammatory stomach lesions induced by HCl/EtOH or aspirin by up to 73 or 78%, as also shown for the standard compound ranitidine (RT), a histamine-2 receptor antagonist clinically prescribed for the treatment of peptic ulcer disease and gastroesophageal reflux [25] (Figures 1(a) and 1(b)). Inflammatory cell infiltration triggered by aspirin was also completely reduced after AQCA administration (3 and 30 mg/kg) (Figure 1(b)(B)), according to measuring the activity of MPO, a key inflammatory enzyme secreted by activated neutrophils, macrophages, and microglia, producing highly reactive oxygen species to cause additional damage [26] and subsequently mucosal damage of stomach tissue was also clearly reduced in AQCA treated group (30 mg/kg) (Figure 1(c)), implying that this compound suppresses gastric inflammation regardless of the stimulatory agents. Importantly, AQCA (3 and 30 mg/kg) significantly suppressed the formation of ear edema triggered by arachidonic acid, an active pain-inducing substrate of the prostaglandin E_2_- (PGE_2_-) generating enzyme COX [27], similar to the activity of the standard drug indomethacin with known COX-inhibitory properties (Figure 2(a)), indicating that AQCA can block COX-induced inflammatory responses by either direct suppression of COX activity or indirect inhibition of COX expression pathway. In agreement with this, acetic acid-induced writhing was also significantly inhibited by AQCA (30 and 60 mg/kg), by up to 57 and 72%, respectively (Figure 2(b)), whereas indomethacin (10 mg/kg) potently suppressed writhing by 85%, implying that COX suppression is one of the inhibitory mechanisms of AQCA. These results strongly suggest that anthraquinones such as AQCA are effective when administered orally. Indeed, orally administered emodin (3-methyl-1,6,8-trihydroxyanthraquinone), one of the active components of the root and rhizome of* Rheum palmatum* that has been used for more than 2,000 years in China, was found to exert inhibitory activities on metabolic disorders in diet-induced obese mice [28]. Rhein (4,5-dihydroxyanthraquinone-2-carboxylic acid), another major anthraquinone compound from the same plant, has been demonstrated to ameliorate hepatic steatosis associated with nonalcoholic fatty liver disease [29]. Nonetheless, as there is insufficient evidence to fully explain the* in vivo* curative activity of anthraquinone, a variety of* in vivo* models must be employed to further investigate the value of anthraquinones as functional food ingredients.

Although anthraquinones are abundant components of numerous edible sources, acute toxicity tests were considered necessary to evaluate the* in vivo* pharmacological activity of AQCA. For this, 1 g/kg of AQCA was orally administered, and various toxicological parameters were examined. As shown in Figure 3, AQCA did not appear to induce any side effects, since there were no alterations in body and organ weights (Figures 3(a) and 3(b)), no gastric irritation unlike with aspirin (300 mg/kg) orally administered (Figure 3(c)), and no change in liver toxicity parameters such as aspartate aminotransferase (AST), alanine transaminase (ALT), or cholesterol (Figure 3(d)). These results strongly suggest that AQCA exerts specific pharmacological activity without unexpected side effects. There are few reports on acute toxicity testing performed with a single anthraquinone compound; however, our data (Figure 3) indicate that AQCA is a safe and effective nutraceuticals.

Our* in vivo* efficacy data regarding arachidonic acid-induced ear edema (Figure 2(a)) and acetic acid-induced abdominal writhing (Figure 2(b)) suggest that AQCA-mediated anti-inflammatory activity is related to COX or a COX-linked pathway. Therefore, we examined whether AQCA suppressed the expression of COX-2, which is closely related to inflammatory symptoms. As shown in Figure 4(a), the increased level of COX-2 in the stomach of HCl/EtOH-treated mice was dose-dependently reduced by AQCA treatment. Considering that ranitidine also suppresses COX-2 mRNA expression (Figure 4(a)), it seems likely that HCl/EtOH-induced tissue damage triggers cellular COX-2 expression. Indeed, we previously confirmed the pathophysiological role of COX-2 in HCl/EtOH-induced gastritis conditions through the ameliorative effects of treatment with the selective COX-2 inhibitor rofecoxib [30] and the flavonoid kaempferol, which also inhibits COX-2 expression (Dung et al., submitted). Further supporting these findings, AQCA effectively inhibited the expression of iNOS, TNF-*α*, and COX-2 in LPS-treated RAW264.7 cells (Figure 4(b)) and suppressed the level of COX-2 protein under the same conditions (Figure 4(c)), implying that AQCA primarily suppresses the expression of inflammatory mediators at the transcriptional level. Indeed, both NF-*κ*B and AP-1 activities, as assessed by luciferase assay [31], under the stimulation with PMA, a known stimulator activating various inflammatory signaling enzymes such as PKC, JNK, p38, IKK, IRAK1 [32], were significantly reduced by 50 *μ*M of AQCA (Figure 4(d)), indicating activity through the signaling pathway for NF-*κ*B and AP-1 activation.

Accumulated results in our laboratory suggest that Src, Syk, IRAK1, IRAK4, extracellular signal-regulated kinases (ERK), p38, and JNK are important upstream signaling enzymes for NF-*κ*B and AP-1 activation in LPS-treated macrophages [33–36]. In addition, these enzymes were also identified in various inflammatory tissues such as HCl/EtOH-treated stomachs, LPS/caerulein-treated pancreases, and LPS/D-galactosamine-treated livers [34, 37], indicating that these enzymes contribute to* in vivo* inflammatory processes in addition to* in vitro* events. On the basis of this information, we investigated whether AQCA regulated the activation of upstream inflammatory signaling enzymes. As expected, the phosphorylation of p38, Src, and Syk and the degradation of IRAK1 were strikingly and significantly inhibited in both HCl/EtOH-treated and aspirin-treated stomachs of mice that were orally administered AQCA (Figures 5(a) and 5(b)). In agreement with this, inhibitors of p38 [SB203580 (SB)], Src (PP2), and Syk [piceatannol (Picea)] also suppressed the expression of TNF-*α*, iNOS, and COX-2, similar to the response to AQCA (Figure 5(c)). In addition, numerous reports have suggested roles of p38, Src, Syk, and IRAK1 in NF-*κ*B and AP-1 activation [38–41]. Early I*κ*B*α* phosphorylation (at 5 min) in LPS-treated RAW264.7 cells associated with the activation of early NF-*κ*B is tightly linked to Syk phosphorylation [42]. It was previously reported that p38 acts as a positive player regulating macrophage-mediated inflammation via activation of AP-1 [40]. Src is regarded as a strong activator of NF-*κ*B induced by prooxidants in Toll-like receptor 4 (TLR4) signaling. Additionally, early rearrangement of the actin cytoskeleton under LPS stimulation is linked to the activation of NF-*κ*B via upregulation of Src activity [43]. It has also been revealed that IRAK1 stimulates both NF-*κ*B and AP-1 pathways in LPS-stimulated macrophages [44]. Taken together, these findings clearly suggest that suppression of these enzymes by AQCA contributes to its anti-inflammatory actions. Nonetheless, considering that the phosphorylation of p38, Src, and Syk and the degradation of IRAK1 are managed by their upstream events including the activation of MKK3/6, actin polymerization, and E3 ligase activity [39, 40, 43], one of these or some of them could be targeted by this compound. Moreover, whether nociceptive activities induced by both arachidonic acid and acetic acid are managed by IRAK1 has not yet been explored. Therefore, we will further identify and validate on the exact target(s) of AQCA by employing kinase assay and overexpression work with full-length gene of putative target enzyme. Additionally, role of IRAK1 in acute pain conditions will be examined.

In summary, the results of this study demonstrate that AQCA is clearly able to inhibit various* in vivo* inflammatory and nociceptive symptoms without altering acute toxicity parameters such as body and organ weight, gastric irritation, and serum liver toxicity parameters. AQCA is capable of downregulating the levels of inflammatory gene expression by blockade of Src, Syk, p38, JNK, and IRAK1, thus blocking NF-*κ*B and AP-1 activation. Since AQCA is one of the major components of numerous vegetables and fruits, we propose that AQCA or AQCA-rich nutraceuticals be developed as functional foods with anti-inflammatory properties.

## Figures and Tables

**Table 1:** PCR primers used in this study.

Name		Sequence (5′ to 3′)
*Real-time PCR*		
iNOS	F	GGAGCCTTTAGACCTCAACAGA
	R	TGAACGAGGAGGGTGGTG
TNF-*α*	F	TGCCTATGTCTCAGCCTCTTC
	R	GAGGCCATTTGGGAACTTCT
COX-2	F	GGGAGTCTGGAACATTGTGAA
	R	GCACATTGTAAGTAGGTGGACTGT
GAPDH	F	CAATGAATACGGCTACAGCAAC
	R	AGGGAGATGCTCAGTGTTGG

*Semiquantitative PCR*		
COX-2	F	CACTACATCCTGACCCACTT
	R	ATGCTCCTGCTTGAGTATGT
GAPDH	F	CACTCACGGCAAATTCAACGGCAC
	R	GACTCCACGACATACTCAGCAC

## Abbreviations

AQCA: Anthraquinone-2-carboxylic acid
NF-*κ*B: Nuclear factor-*κ*B
AP-1: Activator protein-1
JNK: c-Jun N-terminal kinase
Syk: Spleen tyrosine kinase
I*κ*B*α*: Inhibitor of *κ*B*α*
IRAK4: Interleukin-1 receptor-associated kinase 4
EtOH: Ethanol
COX-2: Cyclooxygenase-2
LPS: Lipopolysaccharide.
